# Association of Insulin-like growth factor binding protein 2 genotypes with growth, carcass and meat quality traits in pigs

**DOI:** 10.1186/s40781-015-0063-3

**Published:** 2015-09-03

**Authors:** Sombat Prasongsook, Igseo Choi, Ronald O. Bates, Nancy E. Raney, Catherine W. Ernst, Sornthep Tumwasorn

**Affiliations:** Department of Animal Science, Kasetsart University, Bangkok, 10900 Thailand; Department of Animal Science, Michigan State University, East Lansing, MI 48824 USA; Animal Parasitic Diseases Laboratory, ARS, USDA, Beltsville, MD 20705 USA

**Keywords:** *IGFBP2*, Growth, Meat quality, Pig

## Abstract

**Background:**

This study was conducted to investigate the potential association of variation in the insulin-like growth factor binding protein 2 (*IGFBP2*) gene with growth, carcass and meat quality traits in pigs. *IGFBP2* is a member of the insulin-like growth factor binding protein family that is involved in regulating growth, and it maps to a region of pig chromosome 15 containing significant quantitative trait loci that affect economically important trait phenotypes.

**Results:**

An *IGFBP2* polymorphism was identified in the Michigan State University (MSU) Duroc × Pietrain F_2_ resource population (*n* = 408), and pigs were genotyped by *Msp*I PCR-RFLP. Subsequently, a Duroc pig population from the National Swine Registry, USA, (*n* = 326) was genotyped using an Illumina Golden Gate assay. The *IGFBP2* genotypic frequencies among the MSU resource population pigs were 3.43, 47.06 and 49.51 % for the AA, AB and BB genotypes, respectively. The genotypic frequencies for the Duroc pigs were 9.82, 47.85, and 42.33 % for the AA, AB and BB genotypes, respectively. Genotype effects (*P* < 0.05) were found in the MSU resource population for backfat thickness at 10^th^ rib and last rib as determined by ultrasound at 10, 13, 16 and 19 weeks of age, ADG from 10 to 22 weeks of age, and age to reach 105 kg. A genotype effect (*P* < 0.05) was also found for off test *Longissimus* muscle area in the Duroc population. Significant effects of *IGFBP2* genotype (*P* < 0.05) were found for drip loss, 24 h postmortem pH, pH decline from 45 min to 24 h postmortem, subjective color score, CIE L* and b*, Warner-Bratzler shear force, and sensory panel scores for juiciness, tenderness, connective tissue and overall tenderness in MSU resource population pigs. Genotype effects (*P* < 0.05) were found for 45-min pH, CIE L* and color score in the Duroc population.

**Conclusions:**

Results of this study revealed associations of the *IGFBP2* genotypes with growth, carcass and meat quality traits in pigs. The results indicate *IGFBP2* as a potential candidate gene for growth rate, backfat thickness, loin muscle area and some pork quality traits.

## Background

For a long time, pig breeding programs have focused mainly on reducing the costs. Selection has been aimed at increasing litter size and weight gain, decreasing backfat thickness and improving feed conversion. Now, breeding goals have begun to change and are directed much more toward retail carcass yield and meat quality because of the high economic value of these traits. Genetic improvement for valuable cuts of appropriate quality requires estimates of genetic parameters [1]. The consumption of pork has also changed from quantity to quality with the living standard of city and country residents heightened, resulting in high quality pork becoming one of the main objectives that breeders and producers pursue in breeding.

Many fattening pigs are produced through terminal crossing systems, and the Duroc or Pietrain breeds are commonly used as terminal sires. In general, pigs of the Duroc breed have been found to grow faster, but also to have more backfat than other breeds [2]. The Pietrain breed has also been used for terminal sires, and they have been shown to be leaner with a slower rate of fatty tissue deposition when compared to other breeds [3]. Molecular genetics is a popular tool used in applied science as additional information to selection, yielding more accuracy and faster genetic response to selection [4]. The development of a selection criterion for swine that can be measured early in life and can accurately predict future growth would aid in selection by allowing producers to decrease production cost and time.

The insulin-like growth factor binding protein 2 (*IGFBP2*) is a member of the IGFBP family. Previous studies reported the association of the *IGFBP2* gene with some carcass and body composition traits in farm animals [5–7]. Several significant QTL have been identified on *Sus scrofa* chromosome 15 (SSC15) in the region where *IGBFP2* is located with effects on meat quality traits [8, 9]. Moreover, Edwards et al. [10] also found significant QTL affecting meat color and tenderness on SSC15 in the MSU Duroc × Pietrain F_2_ resource population. In addition, Wang et al. [7] reported the association of an *IGFBP2* polymorphism defined using the PCR-SSCP technique, with production performance in a Lantang × Landrace pig population, and they found that different *IGFBP2* haplotypes were associated with meat color and marbling. For growth traits, a previous study found a significant QTL on SSC15 with effect on average daily gain from birth to 70 days in a crossbred wild boar × Large White pig population [11]. However, research on the association of *IGFBP2* polymorphisms with carcass and meat quality traits in pigs is limited. Consequently, *IGFBP2* has been selected as a candidate gene for further study on its potential effects.

## Methods

### Experimental populations and management

#### MSU Duroc × Pietrain resource population

The Michigan State University (MSU) Duroc × Pietrain resource population was used in this study. This population was established by crossing 4 Duroc sires with 15 Pietrain dams (F_0_), and 6 F_1_ sires and 50 F_1_ dams were retained to propagate the F_2_ generation. The F_1_ pigs were intercrossed and 408 F_2_ pigs were used in this study. Pigs were weaned at 16 to 25 days of age and then sorted into nursery pens by sex and weight. At 10 weeks of age, F_2_ pigs were placed into finishing pens. All F_2_ pigs had *ad libitum* access to feed and water, and commercial corn-soybean-based diets that met or exceeded all NRC requirements [12]. Further details of the population and animal management are found in Edwards et al. [13].

#### Commercial Duroc population

A purebred Duroc population including pigs from three herds obtained from the National Swine Registry (NSR; http://www.nationalswine.com) in the United States was used to further investigate the effect of the *IGFBP2* polymorphism on growth and meat quality traits. Three sire families were sampled per herd with approximately 40 pigs per sire family. Sires were chosen to represent a cross-section of the Duroc sire families within the USA. In each herd, pigs were removed from growth test as they approached 113 kg, weighed and had 10^th^ rib backfat thickness and loin muscle area estimated using B-mode ultrasound. Further details on the population can be found in Choi et al. [14].

### Phenotypic measurements

#### MSU Duroc × Pietrain F_2_ resource population

Phenotypic data for 25 growth traits for 408 F_2_ pigs from the MSU resource population was used for this study. Body weight was measured at birth, weaning, 6, 10, 13, 16, 19 and 22 weeks of age, and average daily gain from 10 to 22 weeks of age (ADG) and days to 105 kg were calculated. B-mode ultrasound (Pie Medical 200SLC, Classic Medical Supply, Inc., Tequesta, FL, USA.) estimates of 10^th^ backfat thickness (BF10), last rib backfat thickness (LRF), and *Longissimus* muscle area (LMA) were recorded at 10, 13, 16, 19 and 22 weeks of age. Collection of phenotypic data was previously described in Edwards et al. [13].

When pigs reached market weight (113.03 ± 8.69 kg), they were transported to one of 2 abattoirs, either the Michigan State University Meat Laboratory (East Lansing, MI) or a small federally inspected plant in western Michigan (DeVries Meats, Coopersville, MI). All pigs were fasted overnight but had *ad libitum* access to water. Postmortem carcass traits recorded included hot carcass weight, *Longissimus* muscle (LM) pH and temperature at 45 min (45-min pH and 45-min temp), LM pH and temperature at 24 h (24-h pH and 24-h temp), dressing percent, pH decline from 45 min to 24 h postmortem (pH decline), first rib backfat thickness, last rib backfat thickness, 10^th^ rib backfat thickness, last lumbar vertebra backfat thickness, number of ribs, carcass length, *Longissimus* muscle area at the 10^th^ rib (LMA) and primal cut weights (ham, trimmed loin, picnic shoulder, Boston shoulder, belly and spareribs). Meat quality measurements included subjective color score, firmness score, marbling score, objective color values of CIE L* (lightness), a* (redness) and b* (yellowness) measured using a Minolta CR-310 colorimeter (Ramsey, NJ), drip loss, Warner-Bratzler shear force (WBS) and sensory panel evaluation (juiciness, muscle fiber and overall tenderness, connective tissue, and off-flavor). Further details of phenotypic data collection are found in Edwards et al. [10].

#### Commercial Duroc population

Three growth traits were recorded for pigs from the Duroc population including 10^th^ rib backfat thickness and *Longissimus* muscle area adjusted to 113 kg live weight, and days of age to reach 113 kg. Adjustments were calculated based on the NSIF guidelines [15].

Soon after growth test completion, pigs were harvested through a commercial packing plant where meat quality traits were recorded. The meat quality traits were 45-min pH, 24-h pH, pH decline, objective color (CIE L*), subjective color score and marbling score. Further details of phenotypic data collection are found in Choi et al. [14].

### Genotypic data collection

#### MSU Duroc × Pietrain F_2_ resource population

### PCR-RFLP assays

Genomic DNA was isolated from venous blood collected in EDTA. The PCR was carried out with 10 ng of genomic DNA. Primers (forward 5′-GGTCTGATTGGAGGG GT GT-3′; reverse 5′-AGCCAAGGAGAAATGTGAA GG-3′) were designed to amplify a 245 bp fragment of intron 2 of the porcine *IGFBP2* gene. Identity of this fragment was confirmed by DNA sequencing (ABI PRISM® 3100 Genetic Analyzer, Applied Biosystems, Foster City, CA, USA), and sequence of the sequence tagged site (STS) was submitted to the NCBI database (GenBank Accession No. BV727778).

The PCR was performed in a final volume of 10 *μ*L containing 1 *μ*L of genomic DNA (10 ng/*μ*L), 1 *μ*L of each primer (5 *μ*M), 0.1 *μ*L of deoxynucleotide triphosphates (25 *μ*M) mixture, 0.6 *μ*L of MgCl_2_ (25 m*M*), 0.1 *μ*L of DNA polymerase (5 U/*μ*L), 1 *μ*L of 10X reaction buffer and 5.2 *μ*L of water on a PTC-200 thermal cycler (MJ research, Watertown, MA, USA). The following PCR profile was used: initial denaturation at 94 °C for 3 min; 30 cycles of 94 °C for 1 min (denaturation), 60 °C for 1 min (annealing) and 72 °C for 1 min (extension), and 72 °C for 10 min (final elongation).

**Fig. 1:**
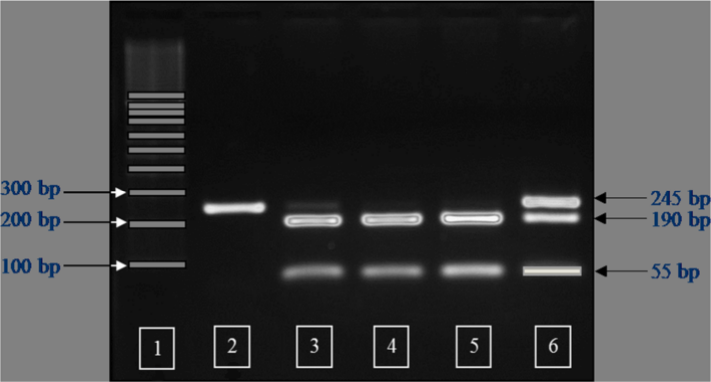
Agarose gel showing the PCR-RFLP pattern for the *IGFBP2* gene amplicons following digestion with *Msp*I. Lane 1, 100 bp molecular weight standards; Lane 2, AA genotype; Lanes 3–5 BB genotype; Lane 6, AB genotype

The *IGFBP2* PCR products were digested using *Msp*I restriction endonuclease at 37 °C overnight. The restriction digests were electrophoresed for 1.5 h at 95 V on a 2.0 % agarose gel with ethidium bromide in 1X TBE buffer. Individual PCR-RFLP fragment sizes were determined by visualizing the band pattern under ultraviolet light. Three genotypes were detected and defined as AA, AB, and BB. The *Msp*I-digested PCR products had fragment sizes of 245 bp for the AA genotype (non *Msp*I recognition site), 190 and 55 bp for the BB genotype, and a combination of 245, 190 and 55 bp for the AB genotype (Fig. 1).

#### Commercial Duroc population

Genomic DNA was isolated from muscle tissue collected at harvest using a PureLink Genomic DNA kit (Invitrogen, Carlsbad, CA, USA). The *IGFBP2* marker identified by PCR-RFLP was included in an Illumina Goldengate 96 plex SNP multiplex custom assay panel [14], and genotyping of *IGFBP2* for pigs from the Duroc population was performed at the MSU Research Technology Support Facility. Genotype segregation to identify pigs as AA, AB or BB was determined using the Illumina Genome Studio software.

### Allele and genotype frequency, and genetic linkage analyses

Allele and genotype frequencies for the *IGFBP2* polymorphism were estimated by the FREQ procedure of SAS (SAS 9.0, SAS Institute, Cary, NC, USA), and Hardy-Weinberg equilibrium (HWE) was tested by *χ*^2^. Genetic linkage analysis of the MSU Duroc × Pietrain resource population was performed using CRIMAP software version 2.4 [16].

### Statistical analysis

The datasets were analyzed by the MIXED procedure of SAS (SAS 9.0, SAS Institute, Cary, NC, USA). The genotype effect within each model was tested with an F-test. If the F-test was significant, t-tests were used to determine significance between estimates of least square means (LSMeans) for the three different genotypes. Significant differences between least squares means of the different genotypes were calculated using a LSMEANS contrast procedure in SAS as follows;

- Additive effect = [AA – BB] / 2
- Dominance effect = AB – [(AA + BB) / 2]

The significance was determined as *P* < 0.05.

#### Growth traits

The statistical analysis model for the MSU Duroc × Pietrain F_2_ resource population included fixed effects of genotype, sex and parity, and random effects of farrowing group, litter and finishing pen nested within farrowing group, as well as covariates appropriate to each trait. The statistical analysis model for the Duroc pig population included genotype, sex and herd as fixed effects and litter as a random effect.

#### Carcass and meat quality traits

The statistical analysis model for the MSU Duroc × Pietrain F_2_ resource population included fixed effects of genotype, sex and slaughter date, random effects of farrowing group and slaughter date nested within farrowing group, and the covariates appropriate to each trait. The statistical analysis model for the Duroc pig population included fixed effects of genotype, sex and slaughter date nested within herd and litter as a random effect.

## Results

### PCR-RFLP analysis and nucleotide sequence validation

**Fig. 2:**
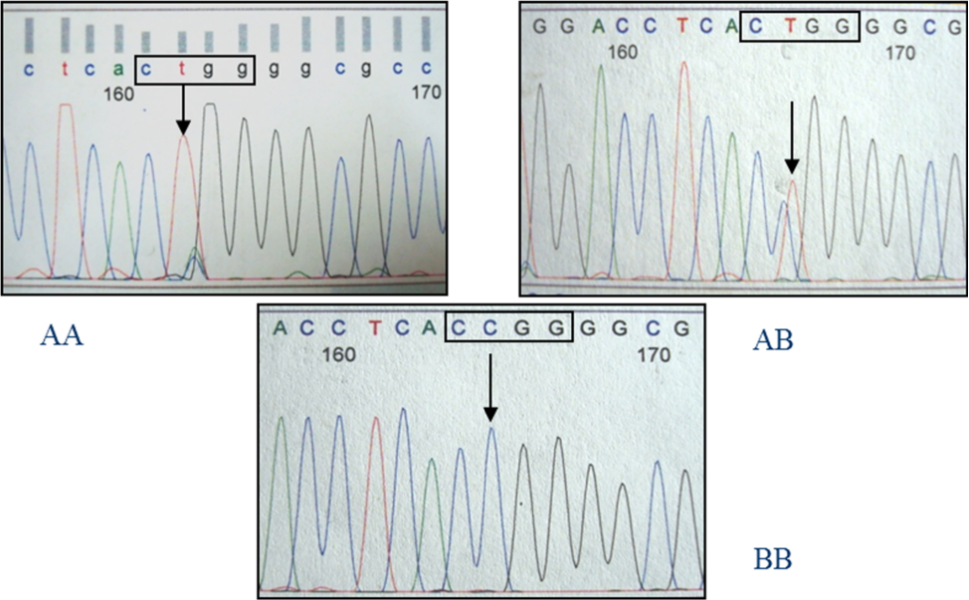
Nucleotide sequences from reverse direction showing the AA, AB and BB *IGFBP2* genotypes (*Msp*I recognition site = CCGG)

The amplification product of the *IGFBP2* intron 2 region was 245 bp in length. Sequencing of this amplicon from multiple individuals showed a C/T single nucleotide polymorphism (SNP). The PCR-RFLP method was developed successfully for SNP genotyping using the restriction endonuclease *Msp*I. Three genotypes were detected and defined as AA, AB, and BB (Fig. 1). Observed fragment patterns were: 245 bp for the AA genotype; 245, 190 and 55 bp for the AB genotype; and 190 and 55 bp for the BB genotype (Fig. 1). DNA sequencing of samples representing each of the three genotypes confirmed the presence of the *Msp*I recognition site (CCGG; Fig. 2). For the AA genotype, nucleotide sequence at the *Msp*I recognition site was CTGG instead of CCGG. Thus, the RFLP fragment pattern on agarose gels showed only uncut fragments for the A allele. Nucleotide sequence for animals with the BB genotype revealed an intact recognition site for *Msp*I (CCGG), and the RFLP pattern on agarose gels showed only the cut fragment pattern. Sequence for animals with the AB genotype indicated the presence of both the C and T nucleotides, and the resulting RFLP pattern on agarose gels showed both uncut and cut fragments. The *IGFBP2* SNP was submitted to the NCBI dbSNP database (ID# ss86353533).

### Allele and genotype frequency, and genetic linkage analyses

The genotypic and allelic frequencies of *IGFBP2* are shown in Table 1 and were in Hardy-Weinberg equilibrium (*χ*^2^ = 0.20 and 1.60; *P* = 0.90 and 0.45 for the MSU Duroc × Pietrain F_2_ resource population and the Duroc pig population, respectively). Genetic linkage analysis using the CRIMAP software version 2.4 [16] estimated the *IGFBP2* map position in the MSU Duroc × Pietrain resource population to be 78.0 cM on pig chromosome 15 (SSC15).

**Table 1:** *IGFBP2* genotype frequencies in the MSU Duroc × Pietrain F_2_ resource population and the Duroc pig population

	MSU resource population		Duroc population	
Genotype	N	Frequency	N	Frequency
AA	14	0.0343	32	0.0982
AB	192	0.4706	156	0.4785
BB	202	0.4951	138	0.4233
Total	408	1	326	1

### Association of *IGFBP2* with growth traits

Least squares means by *IGFBP2* genotype for the growth traits measured in the MSU resource population and the Duroc population are shown in Table 2. Genotype effects (*P* < 0.05) were found for BF10 and LRF at 10, 13, 16 and 19 weeks of age as determined by ultrasound, and ADG from 10 to 22 weeks of age, as well as days to 105 kg for the MSU resource population. For the Duroc pig population, genotype was associated with LMA adjusted to 113 kg (*P* < 0.05).

**Table 2:** Least square means and standard errors for growth traits measured in the MSU pig resource population and the Duroc pig population

Traits	Number	Genotypes		
		AA	AB	BB
MSU resource population				
Birth weight, kg	408	1.50 ± 0.10	1.56 ± 0.04	1.57 ± 0.04
Weaning weight, kg	408	5.31 ± 0.31	5.59 ± 0.16	5.51 ± 0.16
Body weight 6 wk, kg	408	10.83 ± 0.70	11.50 ± 0.48	11.31 ± 0.48
Body weight 10 wk, kg	408	24.59 ± 1.40	25.89 ± 1.05	25.61 ± 1.05
Body weight 13 wk, kg	408	39.88 ± 1.69	41.31 ± 1.10	40.56 ± 1.10
Body weight 16 wk, kg	408	61.27 ± 2.04	61.83 ± 1.10	61.10 ± 1.09
Body weight 19 wk, kg	408	80.46 ± 2.43	80.96 ± 1.42	79.97 ± 1.42
Body weight 22 wk, kg	408	101.95 ± 2.76	100.51 ± 1.24	99.96 ± 1.22
BF10 10 wk, mm	408	7.61 ± 0.44^ab^	8.04 ± 0.19^a^	7.66 ± 0.19^b^
BF10 13 wk, mm	408	9.15 ± 0.72^ab^	10.00 ± 0.47^a^	9.45 ± 0.47^b^
BF10 16 wk, mm	408	12.21 ± 0.90^ab^	12.85 ± 0.52^a^	12.24 ± 0.52^b^
BF10 19 wk, mm	408	16.23 ± 1.27^ab^	16.63 ± 0.74^a^	15.62 ± 0.73^b^
BF10 22 wk, mm	408	20.50 ± 1.53	20.18 ± 0.80	19.57 ± 0.79
LMA 10 wk, cm^2^	408	10.08 ± 0.72	11.04 ± 0.53	10.99 ± 0.53
LMA 13 wk, cm^2^	408	15.67 ± 0.90	16.40 ± 0.60	16.22 ± 0.60
LMA 16 wk, cm^2^	408	23.34 ± 0.99	24.01 ± 0.53	24.17 ± 0.52
LMA 19 wk, cm^2^	408	29.60 ± 1.16	30.92 ± 0.55	30.86 ± 0.54
LMA 22 wk, cm^2^	408	35.26 ± 1.25	36.60 ± 0.60	36.89 ± 0.59
LRF 10 wk, mm	408	6.14 ± 0.29^ab^	6.19 ± 0.12^a^	5.94 ± 0.12^b^
LRF 13 wk, mm	408	7.15 ± 0.38^ab^	7.28 ± 0.16^a^	6.88 ± 0.16^b^
LRF 16 wk, mm	408	9.54 ± 0.59^ab^	9.67 ± 0.27^a^	9.06 ± 0.27^b^
LRF 19 wk, mm	408	12.41 ± 0.89^ab^	12.16 ± 0.49^a^	11.32 ± 0.48^b^
LRF 22 wk, mm	408	14.36 ± 1.07	14.79 ± 0.50	14.22 ± 0.50
ADG 10 to 22 wk, g/day	408	939.37 ± 26.38^a^	891.86 ± 13.22^b^	888.92 ± 13.13^b^
Days to 105 kg, day	408	148.95 ± 3.56^a^	155.41 ± 2.14^b^	157.63 ± 2.01^c^
Duroc pig population				
BF10, mm	277	13.89 ± 0.47	13.47 ± 0.25	13.53 ± 0.27
LMA, cm^2^	277	47.04 ± 0.75^ab^	47.75 ± 0.40^a^	46.40 ± 0.43^b^
Days to 105 kg, day	277	165.57 ± 2.20	167.02 ± 1.14	165.84 ± 1.23

^a, b,c^ Least square means within the same row with different superscripts differ (*P* < 0.05)

As shown in Table 3, the *IGFBP2* alleles exhibited additive effects (*P* < 0.05) for ADG and days to 105 kg, whereas a dominance effect (*P* < 0.05) was found for BF10 at 13 weeks of age in the MSU resource population. For the Duroc pig population, a significant dominance effect was observed for LMA.

**Table 3:** Genetic effects of *IGFBP2* on growth traits for the MSU Duroc × Pietrain F_2_ resource population and the Duroc pig population

Traits	Additive effect	Dominance effect
MSU resource population		
BF10 13 wk, mm	−0.15 ± 0.29	0.69 ± 0.33*
ADG 10 to 22 wk, g/day	25.84 ± 12.03*	−23.04 ± 11.19
Days to 105 kg, day	−4.34 ± 1.51*	2.12 ± 1.75
Duroc pig population		
LMA, cm^2^	−0.32 ± 0.41	1.03 ± 0.52*

Significance was determined as *P* < 0.05 (*), additive effects represent the effect of allele A

### Association of *IGFBP2* genotypes with carcass traits

LSMeans by *IGFBP2* genotypes for carcass characteristics are presented in Table 4. No significant effect of *IGFBP2* genotype was observed for any of the carcass traits (P > 0.05) in the MSU resource population.

**Table 4:** Least square means and standard errors for carcass characteristics of the MSU Duroc × Pietrain F_2_ resource population separated by *IGFBP2* genotypes

Traits	Number	Genotypes		
		AA	AB	BB
Off-farm body weight, kg	408	114.16 ± 2.31	112.78 ± 1.06	113.09 ± 1.06
Hot carcass weight, kg	408	82.56 ± 1.78	82.25 ± 0.79	82.58 ± 0.78
Dressing percent	408	72.30 ± 0.49	72.94 ± 0.22	72.96 ± 0.22
Carcass length, cm	408	78.32 ± 0.58	78.54 ± 0.22	78.62 ± 0.22
First-rib backfat, mm	372	40.35 ± 1.84	41.50 ± 0.99	41.03 ± 0.97
10^th^-rib backfat, mm	433	24.08 ± 1.53	24.33 ± 0.65	23.94 ± 0.63
Last-rib backfat, mm	408	28.93 ± 1.58	28.75 ± 0.88	28.76 ± 0.87
Last-lumbar vertebra backfat, mm	408	22.30 ± 1.58	22.52 ± 0.71	22.53 ± 0.71
LMA at 10^th^ rib, cm^2^	404	39.33 ± 1.06	40.37 ± 0.34	40.73 ± 0.32
Number of ribs in carcass	298	15.08 ± 0.17	14.84 ± 0.06	14.87 ± 0.05
Ham weight, kg	408	9.93 ± 0.13	9.75 ± 0.06	9.81 ± 0.06
Loin weight, kg	408	8.27 ± 0.17	8.24 ± 0.09	8.31 ± 0.09
Boston shoulder weight, kg	408	3.78 ± 0.12	3.80 ± 0.09	3.81 ± 0.09
Picnic shoulder weight, kg	408	3.81 ± 0.12	3.87 ± 0.10	3.89 ± 0.10
Belly weight, kg	408	5.09 ± 0.10	5.05 ± 0.05	5.04 ± 0.05
Spareribs weight, kg	408	1.49 ± 0.04	1.50 ± 0.02	1.50 ± 0.02

### Association of *IGFBP2* genotypes with meat quality traits

LSMeans by *IGFBP2* genotypes for meat quality traits are presented in Table 5. Genotype effects (*P* < 0.05) were found in MSU resource population pigs for drip loss, WBS, 24-h pH, pH decline, subjective color score, CIE L* and b* objective color values, and sensory panel juiciness, tenderness, connective tissue and overall tenderness. Moreover, genotype effects (*P* < 0.05) were found in the Duroc population for 45-min pH, CIE L* and color score.

**Table 5:** Least square means and standard errors for meat quality traits of MSU Duroc × Pietrain F_2_ resource population and Duroc population pigs separated by *IGFBP2* genotypes

Traits	Number	Genotypes		
		AA	AB	BB
MSU resource population				
Drip loss, %	405	2.13 ± 0.31^a^	1.79 ± 0.14^b^	1.44 ± 0.14^b^
WBS, kg	406	3.40 ± 0.18^a^	3.24 ± 0.09^a^	3.03 ± 0.09^b^
45-min pH	405	6.37 ± 0.06	6.35 ± 0.02	6.36 ± 0.02
45-min temp, °C	408	39.33 ± 0.49	39.29 ± 0.40	39.17 ± 0.40
24-h pH	398	5.48 ± 0.03^a^	5.51 ± 0.01 ^b^	5.60 ± 0.01^c^
24-h temp, °C	407	2.71 ± 0.24	2.77 ± 0.19	2.79 ± 0.19
pH decline	395	0.88 ± 0.06^ab^	0.83 ± 0.02^a^	0.77 ± 0.02^b^
Firmness score	408	3.02 ± 0.22	2.86 ± 0.09	2.77 ± 0.09
Marbling score	408	2.87 ± 0.22	2.76 ± 0.10	2.76 ± 0.09
Color score	408	3.06 ± 0.24^ab^	3.05 ± 0.07^a^	3.28 ± 0.06^b^
CIE L* value	381	53.86 ± 0.61^ab^	54.09 ± 0.18^a^	53.37 ± 0.17^b^
CIE a* value	381	16.92 ± 0.26	16.96 ± 0.07	17.14 ± 0.07
CIE b* value	381	9.00 ± 0.20^ab^	9.24 ± 0.06^a^	9.09 ± 0.06^b^
Juiciness score	402	5.09 ± 0.16^ab^	5.20 ± 0.04^a^	5.34 ± 0.04^b^
Tenderness score	406	5.33 ± 0.16^a^	5.48 ± 0.05^a^	5.70 ± 0.04^b^
Connective tissue score	406	6.16 ± 0.10^a^	6.36 ± 0.03^ab^	6.44 ± 0.03^b^
Off-flavor score	406	1.26 ± 0.06	1.16 ± 0.02	1.17 ± 0.02
Overall Tenderness score	406	5.44 ± 0.15^a^	5.55 ± 0.04^a^	5.77 ± 0.04^b^
Duroc pig population				
45-min pH	226	6.41 ± 0.04^ab^	6.44 ± 0.02^a^	6.39 ± 0.02^b^
24-h pH	307	5.84 ± 0.03	5.86 ± 0.02	5.85 ± 0.02
pH decline	223	0.59 ± 0.05	0.60 ± 0.03	0.55 ± 0.03
CIE L*	312	50.94 ± 0.42^ab^	50.80 ± 0.22^a^	51.48 ± 0.22^b^
Color score	325	2.95 ± 0.11^ab^	2.99 ± 0.06^a^	2.87 ± 0.06^b^
Marbling score	326	2.38 ± 0.20	2.23 ± 0.10	2.30 ± 0.10

^a, b, c^Least square means within the same row with different superscripts differ (*P* < 0.05)

As shown in Table 6, the *IGFBP2* alleles exhibited additive effects in the MSU resource population for drip loss, sensory panel tenderness, WBS and sensory panel overall tenderness (*P* < 0.05), and 24-h pH and sensory panel connective tissue (*P* < 0.01).

**Table 6:** Genetic effects of *IGFBP2* on meat quality traits in the MSU Duroc × Pietrain F_2_ resource population

Traits	Additive effect	Dominance effect
Drip loss, %	0.35 ± 0.15*	0.003 ± 0.17
24-h pH	−0.06 ± 0.02**	−0.03 ± 0.02
Tenderness	−0.18 ± 0.08*	−0.04 ± 0.09
WBS, kg	0.18 ± 0.09*	0.01 ± 0.10
Connective tissue	−0.14 ± 0.05**	0.07 ± 0.06
Overall tenderness	−0.16 ± 0.08*	−0.05 ± 0.08

Significance was determined as *P* < 0.05 (*) and *P* < 0.01 (**), respectively, additive effects represent the effect of allele A

## Discussion

Genotyping of pig *IGFBP2* using the *Msp*I PCR-RFLP identified in this study allows clear identification of individuals into AA, AB or BB genotypes. Previous studies have reported polymorphisms at the pig *IGFBP2* locus [5, 7, 17, 18]. Wang et al. [7] identified three pig *IGFBP2* polymorphisms. These researchers then conducted an association study with *IGFBP2* haplotypes and several body composition and meat quality traits for a population of Lantang × Landrace pigs (*n* = 113). The traits evaluated by Wang et al. [7] differed from those evaluated in the present study so it is not possible to directly compare results. In addition, the pig breeds and specific *IGFBP2* polymorphisms evaluated between the two studies differed.

Genetic linkage analysis using the CRIMAP software version 2.4 [16] estimated the *IGFBP2* map position in the MSU resource population to be 78.0 cM on SSC15. Microsatellite markers linked to *IGFBP2* in the MSU resource population were consistent with microsatellites found to be linked to *IGFBP2* by other groups reporting either radiation hybrid mapping [7] or linkage mapping [17] of *IGFBP2* to SSC15. The gene *PRKAG3* (protein kinase, AMP-activated, gamma 3 non-catalytic subunit), which has been shown to be associated with pork quality traits including glycolytic potential, ultimate pH and color [19, 20], is also located in this region of SSC15 [9]. However, it is unclear how closely linked *IGFBP2* and *PRKAG3* are because the two genes have not been located on a common map. It is not currently possible to confirm the physical map position for *IGFBP2* in the pig genome reference sequence (Sus scrofa ver 10.2) because this gene is currently aligned to an unassembled contig (Ensembl Scaffold JH118558.1: 46,034-68,435), and thus it remains unmapped in the most recent genome assembly. Several significant QTL affecting meat quality traits, including color and tenderness, have been identified in the MSU resource population within the SSC15 region containing the *IGFBP2* gene [7, 10, 14]. Wang et al. [7] found *IGFBP2* haplotypes associated with meat color and marbling in a Lantang × Landrace pig population. Therefore, *IGFBP2* maps to a chromosome region containing significant QTL that affect economically important traits and could be a potential candidate gene for further study.

### Association of *IGFBP2* gene with growth traits

Average daily gain (ADG) is one of the most economically important traits and it is used in pig selection indexes. This study found pigs in the MSU resource population with the AA genotype had the fastest weight gain when compared to pigs with the AB or BB genotype. The results were consistent with a report in beef cattle by Pagan [6] who considered *IGFBP2* as a candidate gene and found an association of *IGFBP2* with days on feed. Moreover, a favorable additive effect was detected for ADG (*P* < 0.05) such that the additive effect was approximately 26 g/day per copy of the A allele. Backfat deposition traits (BF10 and LRF), which are important for most pig producers and also pork consumers, were also found to be associated with *IGFBP2* genotype in the MSU resource population when measured at 10, 13, 16 and 19 weeks of age. Pigs with the BB genotype had lower backfat thickness than pigs with the AB genotype. While Wang et al. [7] did not evaluate subcutaneous backfat thickness, they did find an association of *IGFBP2* with leaf fat weight. In addition, Li et al. [5] reported that *IGFBP2* in chicken was associated with multiple traits including abdominal fat. Thus, *IGFBP2* might indirectly affect adipocyte differentiation by controlling IGFs in fat tissue [21].

The end weight goals were different for the MSU resource population and the Duroc population pigs. However, while *IGFBP2* genotype was significantly associated with BF10 and LRF from 10 to 19 weeks of age for the MSU resource population pigs, at 22 weeks of age, BF10 and LRF were not significantly associated with *IGFBP2* genotype. Similarly, off test BF10 was not associated with *IGFBP2* genotype for the Duroc pig population although AB genotype pigs exhibited larger off test LMA than BB genotype pigs. Thus, differences between *IGFBP2* genotype classes for backfat thickness at younger ages may not be related with backfat thickness at market age. However, if *IGFBP2* genotype associations are confirmed, this information could help pig producers through optimization of feeding and management practices.

### Association of *IGFBP2* genotypes with carcass traits

No significant associations were observed between *IGFBP2* genotypes and carcass traits in the MSU Duroc × Pietrain resource population. In contrast, Wang et al. [7] observed that *IGFBP2* haplotypes of Lantang × Landrace pigs were associated with fore-body weight, rear-body weight, bone weight of the rear-body, forelimb weight, rearlimb weight, leaf fat weight, stomach weight, number of ribs and body length. These investigators indicated that their experimental population was too small (*n* = 113) to verify their results. Many of the traits evaluated in the present study differed from those evaluated by Wang et al. [7] and those traits that were in common were not confirmed by our study. In addition, the pig breeds and specific *IGFBP2* polymorphisms evaluated between the two studies differed.

### Association of *IGFBP2* genotypes with meat quality traits

Pork quality is greatly affected by the ultimate pH of the meat reached during the first 24 h after exsanguination, as well as the rate of pH decline. This is due to the anaerobic metabolic decomposition of the glycogen reserves in the muscles that results in the production of lactic acid and a subsequent decline in pH. This process can lead to denaturation of muscle proteins if the decline in pH is too great or if the carcass temperature is too high at even moderately low pH levels, which in turn may result in meat with poor water holding capacity, and in extreme cases, pale, soft and exudative (PSE) meat [22]. Normally, the 24-h pH of muscle drops to between 5.5 and 5.8. If the pH declines very rapidly or very slowly, or if the ultimate pH is very high (above 6.1-6.2) or very low (<5.4), carcass quality characteristics, including water-holding capacity and color, will be significantly affected, potentially leading to PSE or dark, firm and dry (DFD) pork [23]. Therefore, 24-h pH along with drip loss and color are important indicators for optimizing meat quality. Results of this study indicated a significant effect of *IGFBP2* genotype on initial LM pH (45-min pH) in the Duroc pig population such that the BB genotype pigs had an unfavorable 45-min pH. However, 45-min pH was not significantly associated with *IGFBP2* genotype in the MSU resource population. Furthermore, ultimate pH (24-h pH) and pH decline were significantly associated with *IGFBP2* genotype in the MSU resource population. For this study, LM 24-h pH ranged from 5.46 to 5.86, which was consistent with previous studies (ranging from 5.34 to 5.80; [24–26]). The 24-h pH of BB genotype pigs in the MSU resource population (5.60 ± 0.01) was more favorable (*P* < 0.05) than 24-h pH for pigs with either the AA genotype (5.48 ± 0.03) or the AB genotype (5.51 ± 0.01). In addition, pH decline showed similar results in that pigs with the BB genotype had lower (*P* < 0.05) pH decline (0.77 ± 0.02) than those from pigs with the AB genotype (0.83 ± 0.02).

Drip loss was also found to be associated with *IGFBP2* genotypes. High drip loss conditions are caused by denaturation of myosin, whereas the fraction of myosin denatured increases with rapid pH decline and low 24-h pH [27]. Previous studies have shown that 24-h pH has a significant negative correlation with drip loss (range −0.49 to −0.62; [25, 28, 29]). Drip loss is the parameter that indicates the ability of pork to retain moisture (water-holding capacity; WHC) and is an essential quality parameter for both pork producers and consumers. For pork producers, low WHC (high drip loss) implies increased economic losses, and consequently, are interested in optimizing this parameter [30]. The WHC of fresh pork is also known to influence its technological quality, such as processing yield. For pork consumers, low WHC (high drip loss) has an unfavorable impact on the appearance of fresh meat cuts during retail and may influence the sensory quality of the pork [31, 32]. Several factors have been shown to affect the WHC of pork; genotype, particularly the RN^−^ gene (i.e., *PRKAG3*) [33], pre-slaughter stress and stunning method [34], as well as the cooling regime used on the carcass [30]. Results from the present study found pigs with the AB (1.79 ± 0.14 %) and BB (1.44 ± 0.14 %) genotypes to have more desirable (*P* < 0.05) drip loss than pigs with the AA (2.13 ± 0.31 %) genotype, consistent with the results for 24-h pH and pH decline, indicating that pigs with the BB genotype had more favorable phenotypes.

Consumer perceptions of fresh meat products are affected, at least in part, by color and firmness such that darker meat color and more firm products are more desirable. In addition, during the pork chain quality audit, packers reported a 10 % incidence of PSE pork and a 4 % incidence of DFD pork [35]. The association between PSE and DFD quality defects with their respective colors has led the industry to assign visual color scores to carcasses [36], and the use of instrumental color evaluation [37] is of significant interest to the industry because of its speed, consistency of measures, and potential for use as the basis for sorting. Therefore, color is an important economic trait for pork. Various studies have shown an effect of pH on color. Martin et al. [38] evaluated 3,114 pigs from three commercial plants and found 24-h pH explained 40 to 53 % of the variation in fresh pork color. Dransfield et al. [39] evaluated objective color on a population with a wide range of 24-h pH (5.6 to 6.9) and found that 24-h pH explained 53 % of the variation in CIE L* values. DeVol et al. [40] found significant correlations between loin pH and subjective color (*r* = 0.62). Bidner et al. [41] also reported that loin 24-h pH was significantly correlated with CIE L*, a* and b* objective color values (*r* = −0.68, −0.23 and −0.47, respectively). Results of the present study indicated that in the MSU Duroc × Pietrain F_2_ resource population, pigs with the BB genotype had favorable (*P* < 0.05) subjective color scores (3.28 ± 0.06), and objective color values for CIE L* (53.37 ± 0.17) and b* (9.09 ± 0.06). In contrast, for the Duroc pig population, pigs with BB genotype had unfavorable 45-min pH (6.39 ± 0.02), CIE L* (51.48 ± 0.22) and subjective color score (2.87 ± 0.06). The MSU resource population results agreed with Wang et al. [7] who found different *IGFBP2* haplotypes to have significant effects on meat color in Lantang × Landrace pork. Furthermore, the MSU resource population results also tended to follow the results for 24-h pH, because pork color is influenced by residual enzyme activity that will be higher with higher 24-h pH resulting in de-oxidation of oxymyoglobin and consequently darker meat color [32].

Another important trait affecting pork quality is tenderness. Results of this study revealed a significant association of *IGFBP2* genotypes with both a mechanical measure of tenderness (WBS) and a sensory panel assessment of tenderness. Pigs with the *IGFBP2* BB genotype had the lowest (*P* < 0.05) WBS (3.03 ± 0.09 kg) as compared to pigs with AA (3.40 ± 0.18 kg) or AB (3.24 ± 0.09 kg) genotypes indicating that BB pigs had more tender loin muscles. Pigs with the BB genotype also had the most favorable (*P* < 0.05) sensory panel tenderness (5.70 ± 0.04) and overall tenderness (5.77 ± 0.04) values. In addition, sensory panel values for juiciness (5.34 ± 0.04) and connective tissue (6.44 ± 0.03) were more desirable (*P* < 0.05) for pigs with the BB genotype. These observations appear to be consistent with the observations for other meat quality traits in this study. Howard and Lawrie [42] found that the rate of pH decline postmortem was inversely related to meat tenderness. Similarly, Marsh et al. [43] indicated that increased tenderness is observed when pH declines slowly. Thus, the more favorable 24-h pH and pH decline phenotypes for *IGFBP2* BB genotype pigs appear to be consistent with the favorable drip loss, color, WBS and eating quality traits observed in MSU Duroc × Pietrain F_2_ resource population pigs.

## Conclusions

The *IGFBP2* alleles in MSU Duroc × Pietrain F_2_ resource population pigs exhibited additive genetic effects for ADG, whereas a dominance effect was found for BF10 at 13 weeks of age. Pigs with the AA genotype had faster weight gain from 10 to 22 weeks of age than pigs with the AB or BB genotypes. Moreover, pigs with the BB genotype had less backfat thickness than pigs with the AB genotype as determined by ultrasound from 10 to 19 weeks of age. Therefore, pigs with the BB genotype appear to have more desirable backfat thickness, albeit with less desirable rate of weight gain. The effects on growth rate and backfat thickness observed in the resource population were not confirmed in the Duroc population. However, the end weight goals were different for the two populations. The off-test backfat measures were not significantly associated with *IGFBP2* genotype in either population. Thus, differences between *IGFBP2* genotype classes for backfat thickness at younger ages may not be related with backfat thickness at market age. However, if *IGFBP2* genotype associations are confirmed, this information could help pig producers through optimization of feeding and management practices.

Significant associations of *IGFBP2* genotype were found in the MSU Duroc × Pietrain F_2_ resource population for 24-h pH, pH decline, drip loss, subjective color score, CIE L* and b* objective color values, WBS, and sensory panel juiciness, tenderness, overall tenderness and connective tissue. Furthermore, associations were found in the Duroc pig population for CIE L* and color score. Pigs with the BB genotype were found to have more desirable phenotypes for significant traits in the MSU resource population. However, in the Duroc pig population, pigs with the BB genotype had unfavorable phenotypes for significant color traits. The *IGFBP2* alleles exhibited additive effects for drip loss, 24-h pH, WBS, and sensory panel tenderness, overall tenderness and connective tissue in the MSU resource population. The results indicate *IGFBP2* as a potential candidate gene useful for growth rate, backfat thickness, loin muscle area and some pork quality traits in pigs, with the allele considered to be more favorable differing depending on the selection goals for the population.

## Abbreviations

ADG: Average daily gain
BF10: 10^th^ rib backfat thickness
DFD: Dark, firm, and dry
F_0_: Grandparents
F_1_: First filial generation
F_2_: Second filial generation
HWE: Hardy-Weinberg equilibrium
IGFBP2: Insulin-like growth factor binding protein 2
LM: *Longissimus* muscle
LMA: *Longissimus* muscle area
LRF: Last rib backfat thickness
MSU: Michigan State University
NSIF: National Swine Improvement Federation
NSR: National Swine Registry
PCR: Polymerase chain reaction
PSE: Pale, soft and exudative
QTL: Quantitative trait locus
RFLP: Restriction fragment length polymorphism
SNP: Single nucleotide polymorphism
SSC: *Sus scrofa* (porcine) chromosome
USA: United States of America
WBS: Warner-Bratzler shear force
WHC: Water holding capacity
